# The success of the Uttarakhand Cluster: a case study of organisational change towards disability inclusive development

**DOI:** 10.1186/s12913-016-1589-x

**Published:** 2016-08-02

**Authors:** Nathan Grills, Jubin Varghese, Nicole Hughes, Tamara Jolly, Robert Kumar

**Affiliations:** 1 Nossal Institute for Global Health, The University of Melbourne, 161 Barry Street, Carlton, VIC 3053 Australia; 2 Emmanuel Hospital Association, Herbertpur Hospital, Herbertpur, District Dehradun, Uttarakhand 248 142 India; 3 CBM-Nossal Partnership for Disability Inclusive Development, 56 Rutland Rd, Box Hill, VIC 3128 Australia

## Abstract

**Background:**

Persons with disability are often marginalised and excluded from international development efforts. This case study reviews the success of Uttarakhand Cluster of development NGOs in changing organisational behaviour towards being disability inclusive in their development (DID) activities.

**Methods:**

A triangulation of qualitative research methods was used, including key informant interviews, focus group discussions and review of textual data.

**Results:**

The results synthesise data into Kotter’s framework for organisational change, explaining the different stages of change experienced by the Cluster as it moved towards DID. Development of a disability mission, sharing of capacity and resources, and presence of disability champions were key in the organisations' transition towards DID.

**Conclusion:**

This case study demonstrates that the Cluster, a low - cost network, was able to drive organisational change and promote DID.

## Background

In 2011, the World Report of Disability identified that 15 % of the world’s population are people with a disability [1]. In every society across the world, people with disabilities exist and bring with them a unique and diverse set of abilities. As per the UN Convention on the Rights of Persons with Disabilities, persons with disabilities have equal human rights and should be included equitably in all aspects of society [2]. However, despite this convention being signed by 159 countries to include the nearly one billion people living with disabilities [3], exclusion remains an issue across the world, both in developing and developed countries. Persons with disabilities are often marginalised and not supported or encouraged to take part in society [1]. Persons with disabilities are less likely to receive education and less likely to be in paid employment, which contributes to the fact that one out of every five people living in poverty have a disability.

Despite increasing evidence of the link between disability and poverty, persons with disabilities have often been excluded from international development efforts, from services and from supports [4]. Disability Inclusive Development (DID) is a vision for international development to include all people with disabilities and promote equity of opportunity and outcomes for all [5].

Development organisations, largely responsible for actioning development initiatives, need to adapt to promote inclusion for persons with disabilities. This case study reviews the success of the Uttarakhand Cluster of community health and development NGOs (henceforth ‘the Cluster’^1^) in changing organisational behaviour to promote DID across their network. The Cluster is a unique network of community health programs based in the northern Indian state of Uttarakhand. Launched in 2008, as part of the Community Health Global Network,^2^ the cluster now has 50 member organisations covering a catchment area of approximately three million people, who come together for mutual knowledge sharing and program strengthening. The model of community - led clustering of community health programs aims to improve integration with the health system, collaboration and peer-to-peer interaction to enable mutual support, and for exchange of ideas, resources and skills. This enables improved advocacy, power and program effectiveness amongst geographically - focused groups.

Programs within the Cluster had been providing health and development services but had not considered the barriers faced by people with disabilities. Therefore, people with disabilities were being unintentionally excluded from the programs. Although these Cluster programs aimed to reach the poorest or the most marginalised, persons with disabilities were not being considered in activities including health services, water, sanitation and education. In India, people with disabilities have a long history of exclusion, including policy exclusion [6], historical and cultural exclusion [7] and marginalisation [8]. For many cluster programs these issues exist but organisations additionally reported that people with disability were often invisible in the communities, that they felt disability was as a specialist field and that there was considerable difficulty in supporting people with disabilities.

In response to this need, in March 2009, the Cluster decided to focus on disability to raise awareness and promote disability inclusive development – both within their respective programs and as a network. The Cluster formed a partnership with the Nossal Institute for Global Health at the University of Melbourne (which links to the CBM-Nossal partnership) to support this work.

## Aim

The aim of this case study is to describe how a network (the Cluster) can drive change towards DID in its member organisations. This research uses the Cluster as a case study example and describes through its experience how change occurred, and the elements and affects of this change.

## Methods

The success of the Cluster’s progress in DID provided the catalyst to create a case study to capture the lessons of successful promotion of disability inclusion in Uttarakhand. Yin, a founder of formal case study methodological approach, explains how case studies can efficiently explore multiple relationships between multiple variables and are well suited to investigate complex phenomena and the contexts in which they exist [9]. As described, promoting change towards disability inclusion is a complex phenomenon involving interactions between various factors. This case study analysis of a successful example helps us to best understand and describe these phenomena, and helps others understand how and why it was successful.

Yin advised that when elucidating a case study it is helpful to utilise multiple research tools and apply them from different angles [10]. Accordingly, we applied a triangulation of qualitative research methods to document the progress towards DID in this Cluster case study. This involved key informant interviews, a focus group discussion and a document review/analysis. The 50 community health and development programs of the Cluster were the subjects of this research, and were selected on the basis of their membership to the Uttarakhand Cluster.

In trying to understand the how and why of this movement towards DID, a triangulation of the following methods was utilised:

- Five key informant interviews were undertaken with those involved in the leadership of the Cluster’s disability inclusion program. The authors conducted interviews during the period from 2012 to 2014. A focus group discussion (FGD) was undertaken which included the disability coordinators from a selection of the participating NGOs. Two of the authors conducted the FGD exploring how and why disability inclusion had progressed amongst the Cluster members. The researchers developed a FGD guide, and 12 out of the 20 disability coordinators invited to the FGD attended. A time of building trust was important, during which the interviewers and FGD participants shared a social meal. Focus group discussions and key informant interviews were conducted in English. Textual data from publications, feedback forms and communications from the Cluster (internal progress reports and disability awareness literature that was developed) between 2012 and 2015 were reviewed and analysed (please see Appendix for a complete list of the sources that were consulted).

The authors undertook thematic analysis on the data collected. Three meetings of the authors were held to distil the results into a descriptive narrative. Yin outlines that the analysis of case dtuy material should seek to collate and analyse the data into a cohesive narrative to describe the how and why of the phenomenon [10]. Accordingly, following the initial analysis of the key themes of the findings, Kotter’s framework [11] was selected as a useful framework under which to organise the data and tell the narrative of organisational change towards DID. Once the framework was chosen, data, including all texts, were re-analysed using the key themes of Kotter’s framework. Multiple voices were included in this document review, including reports authored by the disability coordinators, external evaluators, project manager and executive facilitator and the summaries of participant feedback forms. All documents were interrogated by reading through and identifying information that was relevant to the different themes.

Kotter describes an eight - step process that leads to organisational change. This process provides both the means and methods that lead to transformation in organisations, and has been used extensively to help organisations develop skill sets to lead change. Although primarily used as a tool in leading change, the authors apply this this helpful process to describe organisational change.

Kotter’s framework was chosen for a number of reasons. Firstly, the key themes identified from the initial thematic analysis matched the eight elements of change identified by Kotter. Secondly, to meet disability inclusive development criteria, organisations needed to change their service delivery, strategic plans and visions, and Kotter’s work identifies the helpful elements that need to be addressed to bring about change in organisations. The authors felt this would be a helpful process to illustrate the possible actions and activities that led to an increase in disability inclusive development in this cluster of organisations of North India.

## Results

Data from triangulated sources of key informant interviews, the FGD and textual reports were collated, and then key themes were identified. The authors identified key themes that largely corresponded to Kotter’s organizational change model [11]. The data were then thematically re-analysed, and all the data were accounted for using the Kotter framework. The triangulation of data confirmed again that this model was appropriate to describe the data generated from the triangulated sources. These thematic headings allowed the authors to present the elements that were essential for promoting organisational change towards DID, and to describe how these impacted the disability inclusion focus of the Cluster. When the data, arranged in themes according to Kotter’s model, was presented to the informants, they largely agreed with the representation. A timeline has also been developed to provide chronological information about the case study (Table 1).

**Table 1:** Chronological order of DID in the Cluster

Date	Activity
2008	Uttarakhand Cluster official formation (17 members)
2009	A few organisations decided to focus on inclusion of persons with disability
2009	Situational analysis
2009 (Oct)	5-day Disability Inclusion Workshop in Uttarakhand (CBM-Nossal)
2009 (Jan-Dec)	DID training tool developed and printed
2010 (3 Dec)	DVD produced and launched at a World Disability Day celebration
2011 (Jul-Sep)	DID Fellowships (Australia) for three Cluster members
2011	Formation of a Cluster Disability Advisory Committee
2012	Massive flood – large response including people with disabilities
2013–2014	Development of Accessibility Fund (Rotary International)
2013	DPO formation commences
2014	Cluster has over 50 member organisations

### Creating a sense of urgency

Informants reported that a sense of urgency was created in the Cluster in 2009, when a decision was made by group members to focus on the inclusion of persons with disabilities. The Cluster in partnership with the Nossal Institute commissioned a situational analysis of disability in Uttarakhand and shared results with the Cluster members at one of the Cluster’s biannual training events called Linking-to-Learn (L2L). Following on from this, a disability inclusion awareness workshop for organisational leaders and staff was undertaken with the support of the CBM-Nossal partnership (October 2009). This workshop sensitised the programs to disability, and the feedback forms highlighted an increased understanding of disability and the urgency for action in their programs. The areas where action was perceived to be urgently needed included understanding about local disability services and agencies, identifying and including those who were excluded, and obtaining resources to enable better responses to persons with disabilities (*see* [5]*, Part B, case study, page 7).* In response, the Cluster commissioned a collaboratively produced DVD that was launched in December 2010 on World Disability Day. Uniquely, this was produced by and starred the members themselves, which helped to ensure local ownership. It was widely used by most members and was shown 955 times (Cluster report) by Cluster programs in the first year, to create disability awareness in their respective areas. The workshops highlighted the need for change within the Cluster, and this promoted a sense of urgency from within the member organisations. This prompted the Cluster to jointly apply for a DID fellowship^3^ and nominate three leaders to visit Australia for the 10-week leadership program (or capacity development program) on DID.

### Formation of a powerful coalition

The initial Cluster, in 2008, was made up of 17 member organisations and by 2014 over 50 members formed ‘a powerful coalition of organisations’ according to one key informant. As a result of the workshop, the Cluster’s attention turned towards DID, with collective commitment to drive a shared vision for DID. Over the seven - year period from 2008 to 2015, 31 Cluster programs nominated disability inclusion focal points. The visibility of the Cluster enabled the many members to engage with an existing local disability network called the Dehradun Disability Forum (DDF). This increased the capacity and resources available to the Cluster and also increased the reach of the DDF. The larger Cluster organisations were able to share support and share disability resources and information with smaller organisations as they moved towards DID. One example was the Anugrah Disability Project helping other Cluster partners to develop disability assessment techniques and disability audit tools, and advising on strategies for the inclusion of people with disability in their programs. Another Cluster member was able to freely offer time and training, which built the capacity of the smaller organisations.

In time, the coalition was built with the formation of a specific Cluster Disability Advisory Committee (CDAC) - a sub-committee of the Cluster board - with the objective of guiding the DID process and promoting disability awareness in the whole Cluster. This group also modelled disability inclusion within CDAC, as the committee included one person with significant disability and two parents of children with profound disabilities. This helped identify the disability leaders within the Cluster.

### Creating vision for change

The Cluster vision for disability inclusion was to improve the quality of life and equal participation for people with disabilities, through ensuring people with disabilities have equal access to and benefit from all health and development activities of the Cluster programs, and to empower persons with disabilities to work for the realisation of their rights through the establishment and networking between self-help groups and Disabled People Organisations (DPO). This shared vision was developed through a network of disability champions named disability coordinators. Each organisation appointed one such person. Initially, according to a published report, 21 organisations participated and they were invited to receive five days of training on disability inclusion organised in kind by the Cluster programs. This built the vision and communicated the vision as each NGO champion developed a plan on how their organisation could improve inclusion.

The self-help groups and groups that were established as a result of this work were new to the mountain areas of Uttarakhand, which previously had not had any formal recognition of these groups. A number of stories explained how the establishment of these groups meant that people with disability had a forum to share ideas and more power to led change in their communities, as they now had a collective voice.

To further develop the vision and develop an evidence - informed approach to DID, the Cluster nominated three health professionals, including one with a disability, for the Australian Leadership Award Fellowships (funded by AusAID). This involved a 10-week placement at the Nossal Institute where they refined their knowledge of disability inclusion, built leadership skills and started the development of a contextualised India practice manual for disability inclusion and a guideline for DPOs. The three disability champions developed and outlined a vision.

In 2011, the Cluster led a local celebration of World Disability Day and promoted, throughout the region, the theme of “Removing barriers, to create an inclusive and accessible society for all”. This demonstrated the powerful coalition growth and cemented the vision of the Cluster to develop organisations that supported and included people with a disability.

### Communicating the vision

On their return from Australia, the champions reported back to their own organisations and the Cluster at an L2L event. This enabled the sharing of the vision, knowledge and experiences. This L2L event included one-day planning session with all organisation leaders to plan how to take the vision of disability inclusion forwards. Therefore, these three champions, through the disability coordinators and cluster program leaders, created a disability vision in most of the 50 Cluster members.

Furthermore, to share the vision, the Cluster developed further resources in Hindi for training and awareness raising, and shared these with multiple agencies. A newsletter was developed, which included regular news, sharing of different issues or barriers faced by people with disabilities and a platform to share the vision for disability inclusion. This was circulated within the Cluster.

On World Disability Day for the past three years many Cluster members organised awareness raising programs in their communities. At least 26 of the 50 members' programs were represented at the events that were co-organised by Cluster members.

### Removing obstacles

Initially a five - day workshop for leaders on disability inclusion was undertaken and the feedback indicated that this successfully addressed myths about disability, helped overcome attitudinal barriers and built the capacity in the leaders to pursue DID.

A key to removing barriers to promoting DID in the Cluster was a tool developed by the Cluster for their members to undertake an organisational assessment. Training on using the tool was provided to the disability coordinators in each Cluster organisation. Reports demonstrated that the tool was widely used. It revealed their lack of sensitivity to people with disabilities, substantial gaps in their supports, and obstacles. The self-assessment led many organisations to develop their own plans to overcome the barriers to promoting inclusion.

Some Cluster members were concerned as to the cost of DID. In response to such concerns, an inclusive WASH course was conducted for the network members and disability coordinators. This demonstrated how simple low - cost strategies could be used to promote DID.

### Creating short - term wins

In the early years the Cluster delivered training programs, developed a disability resource manual, collaboratively produced a disability awareness DVD, and provided training to community health volunteers. Training focussed on understanding the principles of disability inclusion, raising awareness around the need for DID and how to take simple steps to promote disability inclusive development programs. Additionally, disability - specific interventions, such as early identification and referral, generated interest in disability and provided a base for wider disability inclusion activities.

As already indicated, the celebration of World Disability Day provided a quick and low cost win in raising the profile of disability in the Cluster programs and their work areas. Therefore, they were undertaken widely across the Cluster programs from 2010 to 2014.

The Cluster had previously identified accessibility as a barrier to disability inclusion, especially in poor and remote areas. In 2013 and 2014, in response to this, the Cluster created an accessibility fund with the support of Rotary International, to support persons with disabilities to obtain mobility aids, undertake modifications, redesign public places and create inclusive WASH facilities. 21 individuals and organisations benefited from this fund. This allowed Cluster programs to achieve a quick win for DID in their respective NGOs, raise awareness around DID in the villages and provide a base from which to grow DID in their area.

### Building on the change

Building on the Cluster’s work to promote disability awareness, and their numerous network links, new referral pathways were developed and promoted at trainings. The Cluster also developed a disability resource directory that also included the details of the Cluster member who could facilitate a link to that particular resource. As a result, many organisations referred children with disabilities to the new early intervention centre in the local government hospital. This surge in referrals was based on an increase in awareness of referral pathways through the Cluster networking.

A focus is now developing on DPO formation. A one-day coordination meeting was organised by the Cluster in 2013, 2014 and 2015 for the formation and support of DPO. In 2013 the Uttarakhand state disability commissioner also participated in the meeting along with DDF members. The formation of DPOs is being promoted across the Cluster, with many NGOs already working on supporting DPO formation.

The Cluster built on the partnership with the Nossal Institute to collaborate on implementing the Rapid Assessment of Disability (RAD) tool which not only measures disability but, uniquely, identifies the barriers experienced to participation and accessing of human rights. It is this information that helps a program to become disability inclusive. This project received external funding and involved collaboration with the Public Health Foundation India (PHFI). The RAD research project has provided information to make adjustments to existing programs to be inclusive and undertake advocacy initiatives including high-level stakeholder dissemination. This research project represents the Cluster maturing into an organisation that can undertake high-level research to promote DID across India.

### Anchoring the change

A significant event in Uttarakhand that anchored the DID change was an opportunity to demonstrate disability inclusion in the face of a natural disaster. In 2013, a massive flood hit Uttarakhand and around 5000 people were killed [12]. In the aftermath many were left homeless and vulnerable. The Cluster organisations coordinated a large response and placed a special emphasis on people with disabilities. According to one report, “the first question they were asking was, 'What about those with disability?'”. Organisations made lists of people with disabilities and provided extra food packets and specific assistance to support them during the flood relief.

Subsequently, the Cluster, led by one member, initiated and oversaw a disability inclusive disaster risk reduction (DRR). This project prepares communities and people with disability to respond to disasters.

The RAD tool research has also anchored the Cluster’s disability focus by providing an ongoing joint research program. This has kept DID at the forefront of the Cluster’s thinking. The CDAC has been able to steward the disability focus and explore and engage with additional opportunities and programs.

## Discussion

The case study describes a process whereby a group of health and development programs have been intentionally supported to develop a disability inclusive approach to ensure people with disabilities benefit equally from community health programs. This Uttarakhand health and development Cluster began as a small network and has grown into a large network of 50 programs working in health and development. The Cluster has demonstrated success in driving organisational change towards DID. Over a seven year period the member organisations have been able to share knowledge and resources, and challenge one another to change into more disability inclusive organisations. This case study outlines how behaviours have changed, and that the focus on disability inclusive development has changed. The process that has led to DID in the Uttarakhand Cluster can be neatly captured using Kotter’s framework for organisational change.

The clear vision of the Cluster led to a list of objectives that might at first appear overwhelming in the context of competing priorities. Yet, Kreuter concludes that “a well-defined, specific issue” and an “agreed-on vision and goal” were characteristics of a successful collaborative mechanism (p55 Krueter [13]). The Uttarakhand Cluster identified simple sustainable strategies for action, beginning with awareness raising activities, acknowledging that barriers extend beyond physical access to health centre buildings. The activities reflected a twin track approach including a variety of disability specific activities and activities that ensure people with disabilities are included into existing programs. These strategies were facilitated by the allocated focal points within the cluster, ensuring that people with disabilities are actively involved throughout the planning, implementation and evaluation of programs.

The cooperative network was a key component in achieving these ambitious objectives. The idea that a network could be utilised to create and support such a movement is supported by literature outlining the efficacy and universality of networks in public health. Inter-organisation networking among NGOs is becoming increasingly recognised by donors and governments as an effective instrument for change and diffusion of innovation [14–17]. Research on networks reveals that networking between local health organisations increases community awareness and participation [13], provides a forum for synthesising new evidence and ideas, amplifies messages for dissemination, improves efficiency and effectiveness of members through facilitation of learning, provides access to resources, and gives members an opportunity to improve inter-organisational linkages [18]. These known effects of networks help to explain how in this case study the networking approach has successfully created momentum towards DID.

The importance of linking to the external environment (e.g. NGOs, government, international agencies) is evident across many of the stages outlined in the results. The literature supports that strong external linkages are intrinsically important for developing the network and ensuring its impact [13, 18]. In this case, links with the University of Melbourne and CBM for example provided resources, training and support for Cluster-wide change towards disability inclusion. External links to the government and other NGOs also provided opportunities to disseminate ideas and input into local government responses to disability. Undoubtedly the link to the local Dehradun Disability Forum was multiplicative in growing a state-wide response to disability. Links to external players were particularly important in promoting a disability inclusive response to the Uttarakhand flood disaster. The group was able to leverage their existing networks to include a disability response. Although no formal network analysis was undertaken as part of this qualitative study, it seems that networks have been key in facilitating effective diffusion of the innovation of disability inclusion. It would be a useful next step to undertake formal social network analysis, as Valente does in his studies, to map more formally the role that networking played in promoting disability inclusion in these programs.

A common theme across the Kotter steps described in the results was that the leadership who were on board were ‘charismatic’ about DID. This is consistent with diffusion of change and social network theory that outlines the importance of charismatic leaders or “nodes” [14, 19]. For example, Valente shows “how much faster diffusion occurs when initiated by opinion leaders” [20].

These leaders were trained and equipped through visits to Australia, minimal salary supplements and mentorship from CBM - Nossal to organise and execute the activities outlined by Kotter. Through their leadership roles in the Cluster they were in turn able to inspire and equip the leaders of the 50 NGOs. As the results above illustrate, having a passionate and heavily invested leader seems indispensable in bringing about DID across such a wide range of groups. The role of champions is important in moving forward innovations and organisational change [20, 21], however the importance of these champions in advocating for the discriminated is a new and exciting element of the cause for change.

Involving those with disability, or those directly impacted by disability, was not only best practice [2] but also gave credibility and continuity to the Cluster DID movement. The president of the Cluster is the father of a child with severe disability and had a personal experience in disability. The two other champions also had professional and personal experience with disability. Two of the three who travelled to Australia in 2011 were significantly impacted by disability. Their response was not merely career-driven but it was, rather, part of their identity. Their personal experiences also gave them credibility in the eyes of partners, donors and other Cluster members. It widely accepted in the disability field that including those with disability improves the impact of DID programs [22]. As Wright comments, “It is important that the rehabilitation field take advantage of the special knowledge and viewpoints of people who have a disability [23]”. Through the inclusion of people with a lived experience of disability the success and credibility of the Cluster grew.

Despite this progress it has been difficult to keep programs focused on disability inclusion as competing agendas arise. The smaller Cluster programs became too busy with their other activities and inclusion tended to be marginalised. To this end the network was important in being able to continue to plan activities and motivate the programs at particular points in time (L2L trainings, disability specific activities and the inclusion fund). The identification and support of disability champions in each program was an important element to advocate for DID in each program even when the leader was too busy. Thus, the Cluster continues to coordinate follow - up workshops and reflections to build capacity for DID. Keeping this issue at the front and centre of the Cluster has allowed the discussion of DID to become institutionalised as part of the functioning of the Cluster.

### Limitations

The information was gathered form a large number of sources over a long period of time. This made compiling the case study an onerous task, however we believed the time and triangulation of sources was important in the context of change that typically is slow. Organisational change, and subsequent institutionalisation of that change, takes considerable time [24]. As evidenced in the results section, constant pressure for DID was maintained over a five - year period. This was important in embedding the change into the Cluster and constituent Cluster member programs. The first activity was started in 2008 (situational analysis), and various DID activities are continuing to this day.

There are limitations that need to be considered when reviewing the case study data. However, given the paucity of data on how to initiate and sustain successful DID change, we suggest that the case study method is appropriate to describe this complex phenomenon [9]. It generates information on how and why a network approach might be useful in promoting disability inclusion [25]. However, the limitation of any case study methodology is the inability to rigorously generalise to other contexts. When studying networks, as acknowledged by Kelger, the context is important in terms of geography, community values, politics, leadership and network membership [26]. Therefore, caution is needed when applying this model to other contexts. Clearly, further organisational research in the area of DID, including further case studies, need to be completed.

The Kotter model, whilst useful to describe what happened to enable the Cluster to move towards DID, is not completely adequate. It fails to capture the importance of the existing network or relationships that are utilised when developing a new network or focus. In the case of The Cluster its background success in cooperating on health advocacy and health programs was important. The existing relationships that had been formed in the preceding ten years provided a base of trust on which to promote and propagate a significant change in attitude and practice amongst the Cluster members. The Network Function Approach, outlined in Ramalingam et al. [27], is perhaps more sensitive to the pre-existing structures.

The pillars of Building and Amplifying in this approach recognise the importance of pre-existing relationships in the formation or new functional networks.

Whilst persons with disabilities remain marginalised and unable to access development supports throughout the world, it is important that we continue to consider ways in which to support and grow DID. Despite many normative statements about the importance of DID, and many development agencies even requiring DID in projects they fund, there is scant research on how to bring this about [28]. This research contributes to this gap but further research is required to better understand how to bring about organisational change towards DID.

## Conclusion

This case study demonstrates that promoting and sustaining DID can be achieved at low cost through well - supported networks. By generating and maintaining interest in DID, the Cluster was able to drive organisational change towards DID. The identification of disability champions for each program and leveraging of strategic partnerships were both important for the network to promote DID. The DID approaches that were adopted by the Cluster were applied in the face of a natural disaster whereby persons with disabilities were intentionally included in the response. Given its low cost, leveraging networks can be an avenue to promote disability inclusive development.

## Abbreviations

CDAC, Cluster Disability Advisory Committee; CHGN, Community Health Global Network; Cluster, Uttarakhand Community Health Cluster; DID, Disability Inclusive Development; DPO, Disabled Persons' Organisation; DRR, disaster risk reduction; FGD, focus group discussion; L2L, Linking – 2 – Learn; NGO, non-government organisation

## Appendix: List of key resources drawn upon

- ANCP report, CBM engagement tool, literature review on organisational engagement (internal documents)
- Uttarakhand Cluster Awareness DVD (cluster 2012 http://www.chgnukc.org/disability_dvd.html)
- Presentation from disability coordinator for EHA delivered in Australia (2013)
- Summaries from the CHGN Uttarakhand website www.chgnukc.org
- CHGN disability brochure (Michaela Dedek, consultant to the cluster, 2012)
- Case study of disability inclusion in the Cluster (Jolly, 2013)
- ALA Resources/training toolkit (Sprunt and Grills 2011)
- Uttarakhand Cluster programs and resources - The local situational analysis for Uttarakhand (2009)
- The Christian Medical Journal of India “CMJI Disability article” (Kumar, Grills, Verghese, 2010) [29]
- Inclusion fund report (Rotary club, 2012-2014)
- FGD- with disability coordinators and Cluster Board. Facilitate discussion of progress and next steps.

## Footnotes

1 Uttarakhand Community Health Cluster http://www.chgnukc.org/cluster_programs_activities.html#disability

2 Community Health Global Network http://www.chgn.org/

3 AusAID funded Australian Leadership Awards Fellowship in partnership with CBM-Nossal and the Cluster
